# Characterization of bacterial biota in the distal esophagus of Japanese patients with reflux esophagitis and Barrett’s esophagus

**DOI:** 10.1186/1471-2334-13-130

**Published:** 2013-03-11

**Authors:** Ning Liu, Takafumi Ando, Kazuhiro Ishiguro, Osamu Maeda, Osamu Watanabe, Kohei Funasaka, Masanao Nakamura, Ryoji Miyahara, Naoki Ohmiya, Hidemi Goto

**Affiliations:** 1 Department of Gastroenterology and Hepatology, Nagoya University Graduate School of Medicine, 65 Tsuruma-cho, Showa-ku, Nagoya, Japan

**Keywords:** Bacterial biota, 16S rDNA, Esophagitis, Barrett’s esophagus

## Abstract

**Background:**

The distal esophagus harbors a complex bacterial population. We hypothesized that a better understanding of bacterial communities in the esophagus would facilitate understanding of the role of bacteria in esophageal disease. Here, we investigated bacterial composition in the distal esophagus in subjects with a normal esophagus, reflux esophagitis, and Barrett’s esophagus.

**Methods:**

Two biopsy specimens were obtained from the distal esophagus at 1 cm above the gastroesophageal junction under endoscopic examination in 18 patients (6 each with normal esophagus, reflux esophagitis, and Barrett’s esophagus) and used for histological examination and DNA extraction. Fragments of 16S rDNA genes were amplified by PCR using general bacterial primers, and bacterial populations were examined. A third biopsy specimen was taken from the patients with Barrett’s esophagus to histologically confirm the replacement of squamous epithelium with columnar epithelium in the distal esophagus.

**Results:**

Endoscopic diagnoses of normal esophagus, esophagitis, and Barrett’s esophagus were confirmed by histological findings. The total amount of bacterial DNA detected did not significantly differ among groups (p > 0.1). On average, each of the 18 subjects yielded about 350 clones, of which 40 were randomly picked and sequenced. Analysis of 147 16S rDNA sequences from 240 clones of 6 subjects with normal esophagus yielded four phyla, *Proteobacteria* (49%), *Firmicutes* (40%), *Bacteroidetes* (8%), and *Actinobacteria* (3%). Similar analysis of 139 16S rDNA sequences from 240 clones of 6 patients with reflux esophagitis yielded 6 phyla, *Proteobacteria* (43%), *Firmicutes* (33%), *Bacteroidetes* (10%), *Fusobacteria* (10%), *Actinobacteria* (2%), and *TM7* (2%). while that of 138 16S rDNA sequences from 240 clones of 6 cases of Barrett’s esophagus yielded 5 phyla, *Firmicutes* (55%), *Proteobacteria* (20%), *Bacteroidetes* (14%), *Fusobacteria* (9%), and *Actinobacteria* (2%). Thus, microbial communities differed among patients with a normal esophagus, reflux esophagitis and Barrett’s esophagus.

**Conclusions:**

Esophageal bacterial composition differs under conditions of normal esophagus, reflux esophagitis, and Barrett’s esophagus. Diverse bacterial communities may be associated with esophageal disease.

## Background

In western countries, where gastroesophageal reflux disease (GERD) has long been common, the incidence of esophageal adenocarcinoma has increased progressively since the 1970s [1]. Persistent GERD can lead to Barrett’s esophagus [2], in which metaplastic columnar epithelium replaces the normal squamous mucosa, with an accompanying predisposition to esophageal adenocarcinoma [3,4]. The incidence of GERD has recently also increased in Asian countries [5,6], particularly in Japan, with a reported prevalence of esophagitis of 3% in the 1970s [7] versus 14-16% in 2004 [6]. Generally, the cause of esophageal diseases is still speculative. Host genetics may play a key role [8,9], but environmental factors are also likely involved [10]. Colonizing bacteria in all parts of the human digestive tract, from the oral cavity to the anus, are essential to human survival [11-13]. The digestive microbiota is a diverse and dynamic system which has developed a synergistic relationship with its host. Moreover, it also plays a crucial role in the development of the host’s innate and adaptive immune system for the maintenance of a normal physiological environment [14,15]. A complex bacterial biota has been defined in the esophagus [13]. According to an estimate by Pei et al. [13], the bacterial biota in the normal distal esophagus is composed of approximately 6 phyla and 140 species. The determined phyla include *Firmicutes*, *Bacteroides*, *Actinobacteria*, *Proteobacteria*, *Fusobacteria*, and *TM7*, of which *Firmicutes* is the most common, followed by *Bacteroidetes*.

The classical method of bacterial culture excludes a large number of unculturable bacteria, and also misrepresents the abundance of some species due to culture condition-related selection. To overcome these drawbacks, culture-independent methods have been developed, the most common of which involves the amplification and analysis of the 16S rDNA gene in a microbiome [16], on the basis that this gene contains highly conserved regions for the identification of individual species [17]. We hypothesized that increased knowledge of bacterial communities in the distal esophagus would assist our understanding of the role of bacteria in diseases at this site.

Here, to better understand the role of bacteria in diseases of the distal esophagus, we examined bacterial composition at the 16S rDNA gene site in subjects with a normal esophagus, reflux esophagitis, or Barrett’s esophagus using 16S rDNA gene-based culture-independent techniques.

## Methods

### Subjects

Patients presenting to Nagoya University Hospital with gastrointestinal symptoms requiring upper gastrointestinal endoscopy between January 21 2008 and March 2 2009 were eligible for the study. Those who were willing to participate in studies of upper gastrointestinal microbiology and who signed an informed consent form were recruited for this study. Exclusion criteria included the use of antibiotics or PPIs or other acid-reducing treatments in the previous 8 weeks, previous gastric esophageal surgery, active infection of the oral cavity, and HBV, HCV, or HIV infection. Six consecutive patients each with normal esophagus, reflux esophagitis, and Barrett’s esophagus were included (Table 1). Status was confirmed histologically for the morphological features of normal esophagus, esophagitis, and Barrett’s esophagus (identifying intestinal metaplasia). The study was approved by the Ethics Committee of Nagoya University Hospital.

**Table 1:** Patient characteristics

	**Number**	**Sex (M/F)**	**Mean age (min-max)**
Normal subjects	6	2/4	55.5 (41–75)
Reflux esophagitis	6	2/4	75.5 (61–83)
Barrett’s esophagus	6	4/2	73.7 (64–83)

### Specimen processing

During upper gastrointestinal endoscopy, two esophageal biopsies were obtained 1 cm above the gastroesophageal junction, one for DNA extraction and the second for histological examination. A third biopsy specimen was taken from the patients with Barrett’s esophagus to histologically confirm the replacement of squamous with columnar epithelium in the distal esophagus. For each patient, one specimen (approximately 2 mm in diameter) was coded as N1-N6 (normal esophagus), R1-R6 (reflux esophagitis), B1-B6 (Barrett’s esophagus), and randomly assigned a number from 1 to 18 so that researchers who performed subsequent processes were blinded to clinical information. They were then placed in a 1.5-ml test tube and stored at −80°C until processing. DNA was extracted from the biopsy using a tissue DNA extraction kit (QIAamp DNA Mini Kit, Qiagen, Hilden, Germany), and the DNA-enriched fractions were eluted in 200 μl of H_2_O and stored at −20°C.

### Quantitative PCR (qPCR)

The amount of total bacterial DNA was quantified by qPCR using a Stratagene Mx3000P thermal cycler (Agilent Technologies, Santa Clara, CA, USA). Each reaction contained a total volume of 20 μl per well and was performed in triplicate. The qPCR reaction solution contained 0.8 μl (10 μM) of forward and reverse primers, 10 μl SYBR® Premix Ex Taq™ II (2 × conc,TaKaRa, Otsu, Japan), 2 μl (40 ng/μl) of template DNA, and 0.4 ul of ROX™ Reference Dye II (50 × conc,TaKaRa, Otsu, Japan), and was made up to 20 μl with RNase-free water. A 466-bp fragment of the bacterial 16S rDNA gene was amplified using the forward primer 5^′^-TCCTACGGGAGGCAGCAGT-3^′^ and reverse primer 5^′^ –GGACTACCAGGGTATCTAATCCTGTT-3^′^. Thermal cycling conditions were 50°C for 2 minutes and 95°C for 5 minutes, followed by 40 cycles of denaturing at 95°C for 15 seconds, primer annealing at 60°C for 30 seconds, and DNA extension at 72°C for 90 seconds. Finally, a dissociation step was added to qualitatively assess reaction product specificity (temperature raised to 95°C, cooled to 60°C then slowly heated back to 95°C over about 20mins) for melt curve analysis of the PCR products. Plasmids containing cloned 16 s rDNA sequences were prepared into a series of 10-fold dilutions in RNase-free water ranging from 1 × 10^6^ copies to one copy and used as positive control in order to make a standard curve. Quantification of template concentrations was made by linear extrapolation of baseline-subtracted data from the bacterial dilution series standard curve. For each reaction a threshold of luminescence was determined and compared to the standard curve. An equivalent concentration given in colony-forming units could be established for each sample.

### PCR amplification and sequencing of bacterial 16S rDNA

Fragments of 16S rDNA genes were amplified using general bacterial primers (forward, 27 F 5-AGAGTTTGATCCTGGCTCAG-3, and reverse, 1492R 5-GGTTACCTTGTTACGACTT-3). For each PCR, Taq DNA polymerase (Toyobo, Osaka, Japan) was used and mixed in accordance with the manufacturer’s protocol. PCR conditions were 94°C for 2 min, 35 cycles of amplification at 94°C for 30 sec, 52°C for 30 sec, and 72°C for 90 sec, followed by a 10-min extension period at 72°C. PCR products were electrophoresed on an agarose gel, and target DNA fragments were extracted from the agarose gel and purified using a Mono Fas DNA purification kit I (GL Sciences Inc., Tokyo, Japan), ligated with the pGEMT Easy (Promega, Madison, WI, USA) cloning vector, and then used to transform *E. coli* DH5α-competent cells. The cloned inserts underwent sequence analysis using the forward PCR primer. The sequenced clones were analyzed using a standard nucleotide BLAST search of GenBank for homology with known bacterial 16S rDNA sequences. In this study, 16S rDNA sequences with >97% identity with known bacterial species were considered as homologous with that species [18].

## Results

### Quantification of bacterial populations

**Figure 1:**
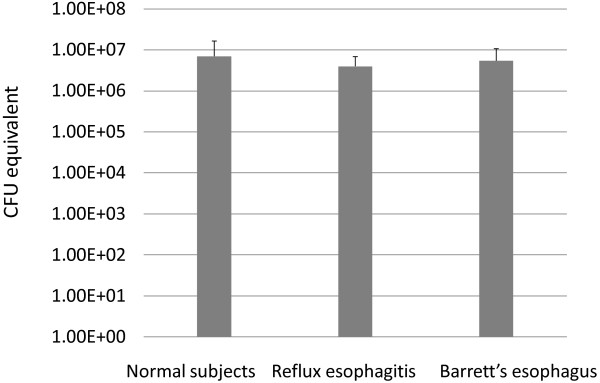
**qPCR analysis of total bacterial load in mucosal biopsy samples.** Mean results are shown for each patient cohort. Error bars denote standard deviation from the mean. Total bacterial load was not significantly different between the groups (p > 0.1; Student’s *t*-test).

Measurement of total bacterial load in mucosal biopsy samples from the distal esophagus in control subjects and patients with reflux esophagitis and Barrett’s esophagus showed high variability among samples, but no significant difference in the total amount of bacterial DNA between the three groups (p > 0.1) (Figure 1).

### Distribution of clones at the phylum and genus levels

Examination of bacterial populations in the distal esophagus by universal 16S rDNA PCR in biopsy samples from 18 subjects revealed an average of about 350 clones in each of the 18 subjects. Of these, 40 were randomly selected and sequenced.

**Figure 2:**
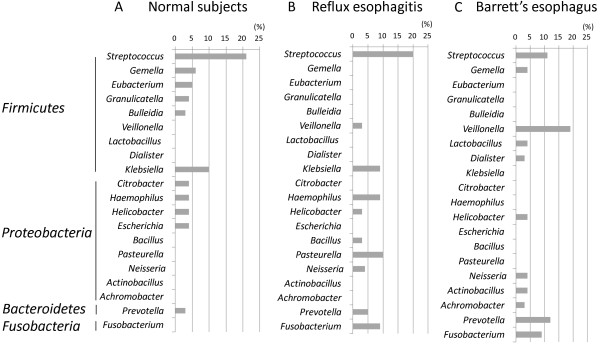
**Distribution of clones at the genus level. A**: Normal subjects. **B**: Patients with reflux esophagitis. **C**: Patients with Barrett’s esophagus. Genus-level distribution of clones in patients with a normal esophagus, reflux esophagitis and Barrett’s esophagus is shown. *Veillonella* was the most prevalent genus in patients with Barrett’s esophagus. *Fusobacterium* was not found in patients with a normal esophagus.

To characterize bacterial populations in normal esophagus, we analyzed bacterial flora from 6 subjects. Two hundred and forty clones (40 clones from each subject) yielded 147 16S rDNA sequences, all of which were classified into 4 phyla. *Proteobacteria* was the most prevalent phylum represented in normal subjects, accounting 49% of clones, followed by *Firmicutes* (40%), *Bacteroidetes* (8%), and *Actinobacteria* (3%) (Table 2). Members of 11 genera (≥3%) were observed, including *Streptococcus* (21%), *Klebsiella* (10%), *Gemella* (6%), *Eubacterium* (5%), *Citrobacter* (4%), *Granulicatella* (4%), *Haemophilus* (4%), *Helicobacter* (4%), *Escherichia* (4%), *Bulleidia* (3%), and *Prevotella* (3%) (Figure 2A).

**Table 2:** Distribution of clones at the phylum and genus levels

	**Normal subject**	**Reflux esophagitis**	**Barrett’s esophagus**
*Actinobacteria*	3%	2%	2%
*Bacteroidetes*	8%	10%	14%
*Firmicutes*	40%	33%	55%
*Fusobacteria*	0%	10%	9%
*Proteobacteria*	49%	43%	20%
*TM7*	0%	2%	0%

no significant difference was detected between the three groups.

To characterize bacterial populations in reflux esophagitis, we analyzed bacterial flora from six patients with reflux esophagitis. Two hundred and forty (40 clones from each subject) clones yielded 139 16S rDNA sequences, all of which were classified into 6 phyla. *Proteobacteria* was the most prevalent phylum of reflux esophagitis, accounting 43% of clones, followed by *Firmicutes* (33%), *Bacteroidetes* (10%), *Fusobacteria* (10%), *Actinobacteria* (2%) and *TM7* (2%) (Table 2). Members of 10 genera (≧3%) were observed including *Streptococcus* (20%), *Pasteurella* (10%), *Klebsiella* (9%), *Fusobacterium* (9%), *Haemophilus* (9%), *Prevotella* (5%), *Neisseria* (4%), *Helicobacter* (3%), *Bacillus* (3%), and *Veillonella* (3%) (Figure 2B).

To characterize bacterial populations in Barrett’s esophagus, we analyzed bacterial flora from six patients with Barrett’s esophagus. Two hundred and forty clones (40 clones from each subject) yielded 138 16S rDNA sequences, all of which were classified into 5 phyla. *Firmicutes* was the most prevalent phylum represented in Barrett’s esophagus, accounting 55% of clones, followed by *Proteobacteria* (20%), *Bacteroidetes* (14%), *Fusobacteria* (9%) and *Actinobacteria* (2%) (Table 2). Members of 11 genera (≥3%) were observed, including *Veillonella* (19%), *Prevotella* (12%), *Streptococcus* (11%), *Fusobacterium* (9%), *Gemella* (4%), *Helicobacter* (4%), *Neisseria* (4%), *Actinobacillus* (4%), *Lactobacillus* (4%), *Dialister* (3%), and *Achromobacter* (3%) (Figure 2C). Compared to patients with normal esophagus or reflux esophagitis, patients with Barrett’s esophagus had a lower percentage of *Streptococcus*.

### Bacteria-positive patient numbers by group

**Figure 3:**
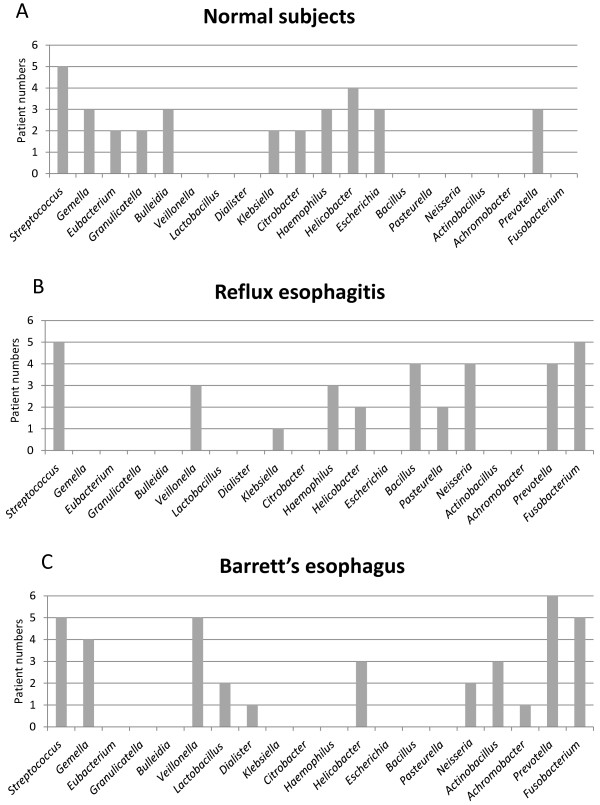
**Bacteria-positive patient numbers. A**: Normal subjects. **B**: Patients with reflux esophagitis. **C**: Patients with Barrett’s esophagus. Y axis shows the number of patients positive for each bacterium at the genus level. *Streptococcus, Provotella* and *Helicobacter* were prevalent in all patients. *Veilonella*, *Neisseria* and *Fusobacterium* were prevalent in patients with reflux esophagitis and Barrett’s esophagus, but were not found in patients with a normal esophagus.

To examine bacterial prevalence in the distal esophagus, we also checked positive patient numbers among the six subjects in each of the normal esophagus, reflux esophagitis, and Barrett’s esophagus groups (Figure 3A, B, C). *Streptococcus, Provotella,* and *Helicobacter* were prevalent in all patients. Interestingly, *Veilonella*, *Neisseria,* and *Fusobacterium* were prevalent in the patients with reflux esophagitis and Barrett’s esophagus, but were not found in the subjects with a normal esophagus. *Streptococcus* was the most prevalent genus in patients with a normal esophagus (5/6), reflux esophagitis (5/6), and Barrett’s esophagus (5/6). *Fusobacterium* was not detected in any subject with a normal esophagus, but was observed in five of six patients each with reflux esophagitis and Barrett’s esophagus. *Helicobacter* was found in four of six subjects with a normal esophagus, two of six with reflux esophagitis, and three of six with Barrett’s esophagus. These findings indicate the presence of a difference in microbial communities between normal esophagus, reflux esophagitis and Barrett’s esophagus.

## Discussion

Here, we analyzed bacteria colonizing the mucosal tissue in Japanese people with a normal esophagus, reflux esophagitis, and Barrett’s esophagus. Our study showed no significant difference in the total amount of bacterial DNA between the three groups (p > 0.1), but unexpected diversity in bacterial populations on the esophageal epithelia in these subjects.

The evolutionary idea that natural selection generally favors the good of the species is currently accepted [19]. This evolutionary history suggests that the bacterial population of the esophagus should remain relatively invariable. Pei Z et al. have reported a complex but conserved bacterial population in the normal distal esophagus [13]. Generally, the relationship between bacteria and the normal esophagus must be balanced, and disruption of this homeostasis may result in esophageal diseases, or esophageal diseases may cause the change in bacterial biota. This prompted us to investigate the relationship between the bacterial biota and esophageal diseases. Our findings suggest the presence of highly complex bacterial populations in the distal esophagus in Japanese subjects, no matter whether they have a normal esophagus, reflux esophagitis, or Barrett’s esophagus. We also found that esophageal bacterial composition differs between these groups. Traditionally, the human esophagus has not been viewed as a hospitable environment for microorganisms because of anatomical structure, and culture-based approaches that have been used to analyze microbiota showed that the esophagus was either sterile or contained only a few transient bacteria [20,21]. However, conventional culturing techniques cannot detect all the bacteria in the gut because of the requirements of an anaerobic and complex environment [22]. The recent use of molecular methods and, in particular, genetic sequencing have revealed a much more diversified flora, of which some are cultivable by traditional techniques whereas many others are not [23]. Pei Z et al. studied bacterial biota in the human distal esophagus using a phylogenetic approach based on 16S rDNA sequences [13]. In the present study, we analyzed bacterial 16S rDNA sequences using a standard nucleotide BLAST search of GenBank for homology with known bacterial 16S rDNA sequences.

Regarding the amount of bacteria in the distal esophagus, we used a real-time PCR method to amplify a 466-bp fragment of the bacterial 16S rDNA gene. Results demonstrated the presence of numerous bacteria in the esophagus, in contrast to previous reports using culture-based approaches [20,21,24]. Interestingly, our study showed no significant difference in the amount of bacteria between groups with a normal esophagus, reflux esophagitis, or Barrett’s esophagus (p > 0.1).

Phylum-level analysis of our present samples revealed that the bacterial communities differed among groups. First, each group had a different number of phyla: populations could be classified into four phyla in patients with normal esophagus, six in those with reflux esophagitis, and five in those with Barrett’s esophagus. Second, phyla composition differed among groups. For example, *Fusobacteria* was found in patients with reflux esophagitis or Barrett’s esophagus but not in those with a normal esophagus. We found differences in composition at the genus level among the normal esophagus, reflux esophagitis, and Barrett’s esophagus groups. We compared not only the distribution of bacterium 16S rDNA gene clone libraries but also the bacteria-positive patient numbers. The most prevalent genus was *Streptococcus* in patients with a normal esophagus or reflux esophagitis, versus *Veillonella* in patients with Barrett’s esophagus. Among other genera, we found that *Fusobacterium* was not detected in normal esophagus but was detected at 9% in both reflux esophagitis and Barrett’s esophagus. A number of other disease-associated differences in genera were noted (Figure 2). These findings therefore show that bacterial populations differ among normal subjects and patients with reflux esophagitis or Barrett’s esophagus at both the phylum and genus levels.

According to an estimate by Ashktrab et al. in African Americans [25], the *H. pylori* positivity was much smaller in patients with esophagitis (4%) than in normal controls (34%). The prevalence of *Helicobacter pylori* (*H. pylori*) infection varies widely by geographic area, age, race and socioeconomic status [26]. Generally, *H. pylori* is a common stomach bacteria among Asian populations [26]. It has been suggested that Asians could be protected against GERD by their high prevalence of *H. pylori* infection [27]. The declining prevalence of *H. pylori* infection due to improved hygiene conditions or use of *H. pylori* eradication therapy might have contributed to the recent increased frequency of reflux esophagitis in Asia [27]. Here, we found *Helicobacter* in four of six subjects with a normal esophagus, two of six with reflux esophagitis, and three of six with Barrett’s esophagus. Allowing for the small number of subjects in each group, we consider that these findings may support the idea that a high prevalence of *H. pylori* infection protects against reflux esophagitis.

Several limitations of our study warrant mention. First, we evaluated bacterial microbiota in the distal esophagus in patients with gastro-esophageal reflux disease (GERD), Barrett’s esophagus, and normal esophageal mucosa using a small data set. Second, the normal subjects were younger than the reflux esophagitis and Barrett’s esophagus patients, which may have affected bacterial variability. Third, we defined species as homologous when their 16S rDNA sequences had >97% identity with known bacterial species. However, comparative studies have clearly revealed the limitations of sequence analysis of this conserved gene and gene product in the determination of relationships at the strain level, for which DNA-DNA reassociation experiments still constitute the superior method [18]. Accordingly, additional study with more refined data collection and an increased number of normal older subjects is required.

## Conclusions

Esophageal bacterial composition differs among subjects with a normal esophagus, reflux esophagitis, and Barrett’s esophagus. Diverse bacterial communities may be associated with esophageal diseases, and comparison of bacterial populations in the esophagus may enhance our understanding of the role of bacteria in esophageal diseases.

## Abbreviations

GERD: Gastroesophageal reflux disease; qPCR: Quantitative PCR.

## Competing interests

The authors declare that they have no competing interests.

## Authors’ contributions

NL, TA, and HG were responsible for the study design and co-ordination. Samples were collected and prepared by TA, KI, OM, OW, KF, MN, RM, and NO. Histological examination was done by TA. DNA extraction and PCR were done by NL. NL analyzed the data. TA drafted the report. All authors participated in critical review of the paper. All authors read and approved the final version of the manuscript.

## Pre-publication history

The pre-publication history for this paper can be accessed here:

http://www.biomedcentral.com/1471-2334/13/130/prepub
